# Nonalcoholic Fatty Liver Disease and Risk of Dementia: A Population-Based Cohort Study

**DOI:** 10.1212/WNL.0000000000200853

**Published:** 2022-08-09

**Authors:** Ying Shang, Linnea Widman, Hannes Hagström

**Affiliations:** From the Department of Medicine (Y.S., H.H.), Huddinge, Karolinska Institutet; Division of Biostatistics (L.W.), Institute of Environmental Medicine, Karolinska Institutet; Division of Hepatology (H.H.), Department of Upper GI, Karolinska University Hospital; and Clinical Epidemiology Unit (H.H.), Department of Medicine, Karolinska Institutet, Stockholm, Sweden.

## Abstract

**Background and Objectives:**

Nonalcoholic fatty liver disease (NAFLD) and dementia share common risk factors including metabolic disorders. However, whether NAFLD is associated with dementia risk is unclear. We investigated the association between NAFLD and dementia risk as well as the role of cardiovascular complications including heart disease and stroke.

**Methods:**

In this population-based matched cohort study, we identified all Swedish patients aged 65 years or older with NAFLD identified from the National Patient Register (NPR) between 1987 and 2016. These were matched with up to 10 reference individuals from the general population on age, sex, and municipality at the year of diagnosis. Incident dementia diagnosis was derived from the NPR or the Cause of Death Register until 2016. Adjusted hazard ratios (aHRs) and 95% CIs were estimated with Cox regression models.

**Results:**

A total of 2,898 patients with NAFLD and 28,357 matched controls were identified (median age at entry, interquartile range [IQR], 70 [8]; 55.1% female). During a median follow-up of 5.5 years (IQR: 8.5 years), 145 (5.0%) patients with NAFLD and 1,291 (4.6%) reference individuals were diagnosed with dementia. Compared with the reference individuals, patients with NAFLD had higher rates of dementia (aHR 1.38, 95% CI 1.10–1.72) and vascular dementia (aHR 1.44, 95% CI 0.96–2.23, *p* = 0.07). Comorbid NAFLD and either heart disease (aHR 1.50 95% 1.08–2.05) or stroke (aHR 2.60 95% CI 1.95–3.47) conferred a greater risk of dementia.

**Discussion:**

NAFLD had a modest association with increased rates of dementia. This was stronger among patients with NAFLD diagnosed with cardiovascular comorbidities.

**Classification of Evidence:**

This study provides Class II evidence that nonalcoholic fatty liver disease is associated with the development of vascular and nonvascular dementia.

Nonalcoholic fatty liver disease (NAFLD) is the most common chronic liver disease, affecting 25% of the global population.^1^ NAFLD is associated with metabolic disorders and is commonly seen as the hepatic manifestation of the metabolic syndrome, and acquiring additional components of the metabolic syndrome, such as type 2 diabetes or hypertension, has been associated with a higher risk of dementia.^2-5^ Although such metabolic disorders are not unique for NAFLD or dementia, it is unclear whether NAFLD contributes to dementia independent of these factors.

Because problems with attention, forgetfulness, and memory have often been reported among patients with NAFLD,^6,7^ a link between NAFLD and poor cognition has been suggested. Notably, studies examining the association between NAFLD and cognitive outcomes have been mostly limited to mild cognitive impairment, with a cross-sectional design.^8-11^ Very few longitudinal observational studies have examined the risk of dementia in NAFLD. One recent cohort study found that elevated liver enzymes were associated with a higher risk of Alzheimer disease (AD) and greater brain atrophy.^12^ Another study reported a higher risk of all-cause dementia in patients with both liver fibrosis due to NAFLD and frailty.^13^ By contrast, other studies failed to find a link between NAFLD and cognitive decline^14^ and incident dementia.^15,16^

The major hypothesis linking NAFLD to cognition is through a vascular pathway.^9^ Indeed, compared with individuals without NAFLD, patients with presumed liver fibrosis due to NAFLD exhibited signs of executive function deficits^11^ and more pronounced white matter lesions.^17^ These are the early markers of functional and structural impairments in vascular dementia.^18^ In addition, the risk of dementia may be further elevated by cardiovascular diseases (CVD) because these conditions are often concomitant, and cardiovascular diseases are the leading cause of death for patients with NAFLD.^19^ However, the possible joint effect of NAFLD and cardiovascular diseases on dementia risk is not well known. Therefore, our aims were to (1) investigate the impact of NAFLD on the risk of dementia and dementia subtypes and (2) examine the role of comorbid cardiovascular disease on these associations.

## Methods

### Study Population

**Figure 1:**
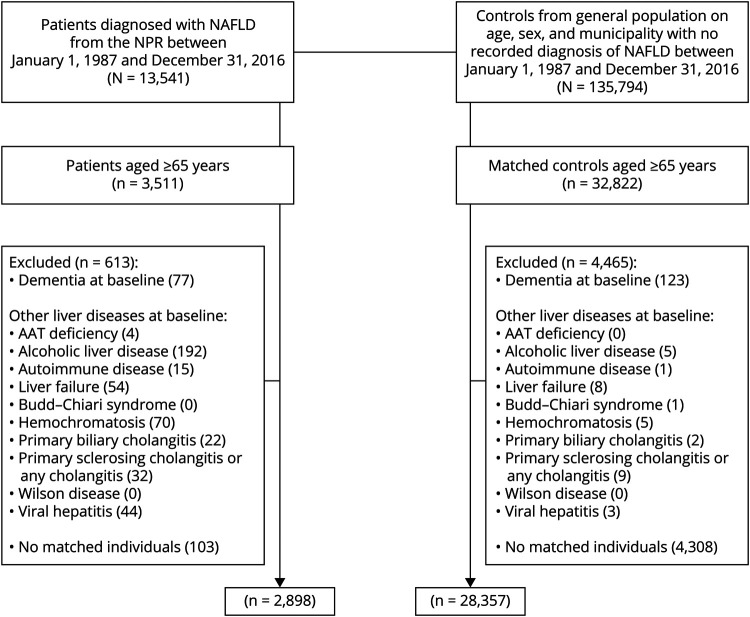
Flowchart of the Study Population AAT-deficiency = Alpha-1 antitrypsin deficiency; NAFLD = nonalcoholic fatty liver disease; NPR = National Patient Register.

We conducted a cohort study of all patients aged 65 years or older with an International Classification of Diseases (ICDs) code corresponding to NAFLD (ICD-9:571.8; ICD-10: K75.8 or K76.0) from the National Patient Register (NPR) between January 1, 1987, and December 31, 2016. The NPR includes data from inpatient care with national coverage since 1987 and includes all visits in specialized outpatient care since 2001. The validity of NPR is regarded as high,^20^ with positive predictive values ranging from 85% to 95% for most chronic diseases including CVD.^20^ The positive predictive value (PPV) of a NAFLD diagnosis from NDR is 89%. Furthermore, the PPVs for dementia diagnosis from NPR and cause of death register (CDR) are 81% and 99%.^21^ We defined the index date as the date of the first NAFLD diagnosis, and the age at diagnosis was obtained for all patients. Each patient with NAFLD was then matched with up to 10 individuals from the general population without a previous diagnosis of NAFLD (matched cohort) randomly selected from the Total Population Register on age, sex, and municipality at the year of diagnosis.^22^ We excluded patients with a diagnosis of NAFLD younger than age 65 years because 80% of NAFLD diagnosis were made since 2001 and the median age of dementia diagnosis in Sweden is 85 years,^21^ meaning that the number of dementia outcomes in younger patients would be low. We further excluded those with prevalent or subclinical dementia and those with other liver diseases on or before the index date both in patients with NAFLD and the matched cohort (eTable1, links.lww.com/WNL/C106, lists the ICD-based definitions of these). Figure 1 shows the flowchart of the study population.

### Study Outcomes and Covariates

Dementia was defined as having an ICD code corresponding to dementia (ICD-9: 290; ICD-10: F00-03, G30-31) from the NPR or the CDR. The CDR is a high-quality data source which contains information on the date of death and underlying and contributing causes of death for all deceased inhabitants of Sweden.^23^ We also identified dementia subtypes, defined as Alzheimer disease (ICD-9: 290.1, 331.0; ICD-10: F00, G30), and vascular dementia (ICD-9: 290.4; ICD-10: F01). Of note, the ICD-9 code of subclinical AD (290.1) was used to exclude AD at baseline, but not to define the outcome.

Age at index date was categorized into 3 groups: 65–74, 75–84, and 85 years or older. Diagnoses of cirrhosis, depression, and relevant metabolic disorders including both type 1 and type 2 diabetes, dyslipidemia, obesity, and hypertension, that all may affect dementia risk, recorded at or before the index date were identified from the NPR. Cardiovascular complications including stroke (ischemic or hemorrhagic) and heart disease (HD) (coronary heart disease, atrial fibrillation, or heart failure) recorded at or before the index date were also collected from the NPR (see eTable 1, links.lww.com/WNL/C106 for ICD codes)*.*

### Statistical Analysis

Baseline characteristics of patients with NAFLD and the matched cohort were compared with chi-square tests for categorical variables and Mann-Whitney U tests for continuous variables. Patients with NAFLD and the matched cohort were followed from the index date to the date of dementia diagnosis, emigration, death, or the end of follow-up (December 31, 2016), whichever came first. We calculated the incidence rate of dementia per 1,000 person-years (PYs) as the number of events divided by the total person-years at risk. Stratified Cox proportional hazard models were used to estimate hazard ratios (HRs) and 95% CIs for incident dementia. To eliminate the effect of other liver diseases on the association between NAFLD and dementia, we censored persons at the date when any other liver disease was diagnosed during follow-up. We also examined the association between NAFLD and dementia subtypes (Alzheimer and vascular dementia). We reported the HRs from 3 models: model 1, crude estimates; model 2 was adjusted for metabolic disorders on index date including diabetes, hypertension, dyslipidemia, and obesity; and model 3 was additionally adjusted for stroke, heart disease, and depression. These models were performed separately for male patients and female patients and for the 3 age groups. The proportional hazard assumptions were tested based on the Schoenfeld residuals. Cumulative incidences of dementia and vascular dementia were computed by cumulative incidence functions for patients with NAFLD and their matched cohort, accounting for nondementia death as a competing risk.^24^

Because stroke and HD are common comorbidities of NAFLD and may modulate the risk of NAFLD on dementia, the interactions between NAFLD and stroke and HD were tested separately. First, we incorporated a cross-product between NAFLD and stroke in the Cox models to determine whether an interaction was present. We also tested the interaction between NAFLD and HD. Second, to assess the magnitude of the risk, we created an indicator variable that combined NAFLD, stroke, and HD, dividing the study population into 8 groups: (1) matched cohort who were free of stroke, HD, and NAFLD (“No disease”); (2) patients with NAFLD who were stroke-free and HD-free (“NAFLD only”); (3) matched cohort who had HD but were stroke-free (“HD only”); (4) matched cohort who had stroke but were HD-free (“Stroke only”); (5) patients with NAFLD and stroke but HD-free (“NAFLD + Stroke”); (6) patients with NAFLD and HD but stroke-free (“NAFLD + HD”); (7) matched cohort with stroke and HD (“Stroke + HD”); and (8) patients with NAFLD, HD, and stroke (“NAFLD + HD + Stroke”). This indicator variable was entered into the Cox regression models adjusted for diabetes, dyslipidemia, obesity, hypertension, and depression.

Two-sided *p* values <0.05 were considered statistically significant, except in the case of interactions analysis, where *p* values <0.10 indicate the presence of a significant multiplicative interaction. All statistical analyses were performed using Stata MP 17.0 (StataCorp).

### Standard Protocol Approvals, Registrations, and Patient Consents

This study was approved by the Regional Ethics Review Board in Stockholm (dnr 2017/1019-31/1). Because this study included analyses of deidentified data, written informed consent from participants was not required.

### Data Availability

Data are subject to personal information protection regulations and are not publicly available. Sharing of anonymized data will be considered on a case-by-case basis on request.

## Results

### Characteristics of the Study Population

We identified 2,898 patients with NAFLD and 28,357 reference individuals aged 65 years or older. The median age for patients with NAFLD and the matched cohort was 70 years (interquartile range [IQR]: 8), with 73% of individuals aged between 65 and 75 years (Table 1). More than half of the study population were female (55.1%). Of all patients with NAFLD, 105 (3.6%) had cirrhosis at baseline. In general, patients with NAFLD were more likely to have metabolic disorders, cardiovascular comorbidities, and depression than the matched cohort (*p* < 0.001 for all).

**Table 1:**
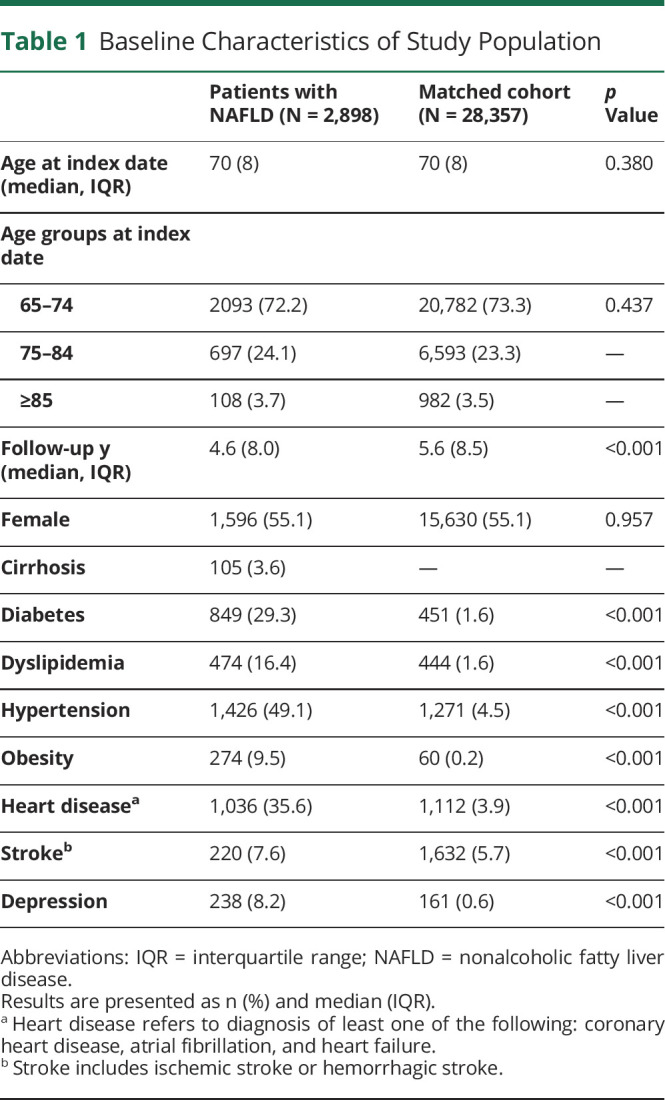
Baseline Characteristics of Study Population

### NAFLD and Dementia Risk

During a median follow-up of 5.5 years (IQR: 8.0) (4.6 years [IQR: 8.0] for patients with NAFLD, 5.6 years [IQR: 8.5] for the matched cohort), 145 (5.0%, 11.5/1,000 PYs) patients with NAFLD and 1,291 (4.6%, 7.9/1,000 PYs) matched cohort developed dementia. This translated to a HR of 1.86 (95% CI 1.55–2.25) for dementia associated with NAFLD relative to the matched cohort (Table 2). The association between NAFLD and dementia was attenuated after adjusting for metabolic disorders (aHR 1.64, 95% CI 1.29–2.07) and further reduced but still statistically significant after additionally adjusting for depression, stroke, and HD (aHR 1.30, 95% CI 1.10–1.72). The risk of dementia was similar across sex (*p* for interaction with sex = 0.44). The risk of dementia was attenuated for patients diagnosed with NAFLD older than age 85 years (aHR 1.08, 95% CI 0.55–2.22), although this subgroup was small (n = 108, 3.7% of the full population, with 12 cases of dementia).

**Table 2:**
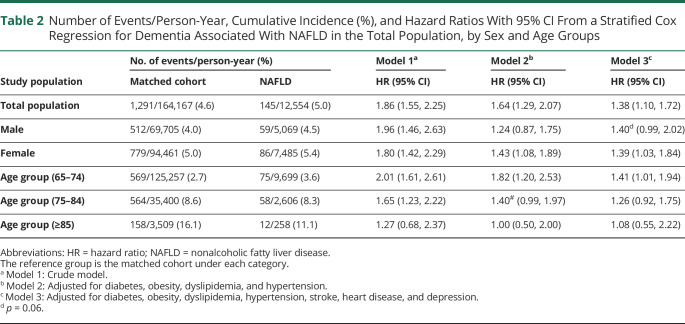
Number of Events/Person-Year, Cumulative Incidence (%), and Hazard Ratios With 95% CI From a Stratified Cox Regression for Dementia Associated With NAFLD in the Total Population, by Sex and Age Groups

Regarding dementia subtypes, Cox regression models suggest that NAFLD is related to a somewhat higher rate of vascular dementia (aHR 1.44, 95% CI 0.96–2.23, *p* = 0.07) but not with the rate of AD (aHR 1.15, 95% CI 0.78–1.70) (Table 3).

**Table 3:**
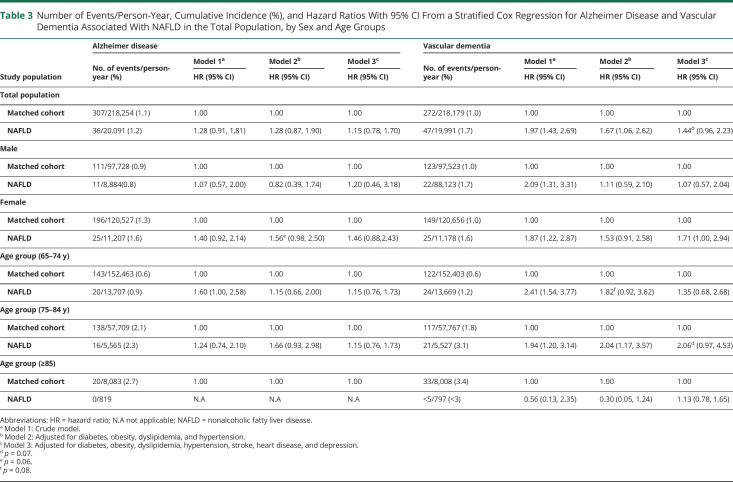
Number of Events/Person-Year, Cumulative Incidence (%), and Hazard Ratios With 95% CI From a Stratified Cox Regression for Alzheimer Disease and Vascular Dementia Associated With NAFLD in the Total Population, by Sex and Age Groups

**Figure 2:**
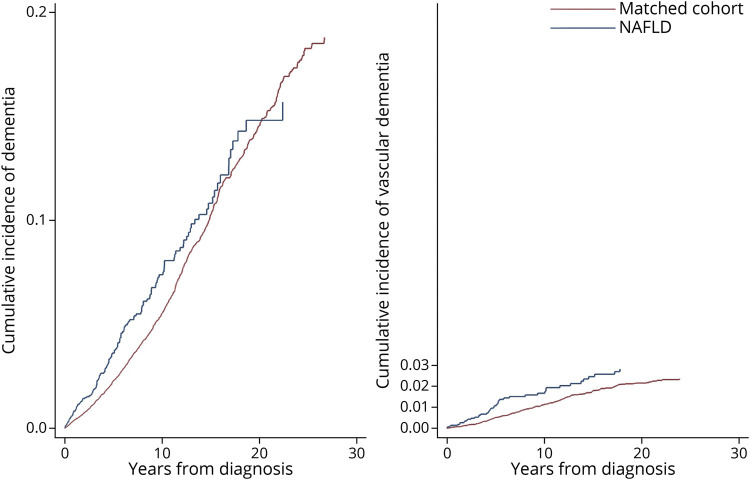
Cumulative Incidence of All-Cause Dementia and Vascular Dementia by NAFLD and Matched Cohort NAFLD = nonalcoholic fatty liver disease.

Cumulative incidences for dementia and vascular dementia for patients with NAFLD and the matched cohort are shown in Figure 2. In general, the probability of dementia was higher for patients with NAFLD than the matched cohort. For example, the 5-year cumulative incidence of all-cause dementia was 3.6% for patients with NAFLD and 2.0% for the matched cohort. Ten years from the index date, 7.5% of patients with NAFLD and 5.5% of the matched cohort developed all-cause dementia. Cumulative incidences across age groups showed that the patients with NAFLD diagnosed at younger than age 85 years had a higher probability of dementia than those diagnosed at age 85 years or older (eFigure 1, links.lww.com/WNL/C106), although the latter subgroup was small (n = 108 patients with NAFLD). Regarding vascular dementia, the 10-year cumulative incidence was 1.9% for patients with NAFLD and 1.1% for the matched cohort.

### Role of Comorbid Heart Disease and Stroke on the NAFLD-Dementia Association

Statistically significant interactions were observed between stroke and NAFLD (*p* = 0.03) but not between HD and NAFLD (*p* = 0.57). Stratified analysis revealed that among patients without stroke, NAFLD was associated with a higher risk of dementia than the matched cohort. Such association was observed in patients without HD, but not in relation to those with stroke or with HD (eTable 2, links.lww.com/WNL/C106)*.* Table 4 presents the joint effect of HD and stroke on the association between NAFLD and dementia. Relative to the matched cohort with neither NAFLD, HD, nor stroke (no disease), those with NAFLD, stroke, or HD alone had higher rates of incident dementia in the fully adjusted model (NAFLD only: aHR 1.42, 95% CI 1.10–1.82; HD only: aHR 1.90, 95% CI 1.43–2.53; stroke only: aHR 2.12, 95% CI 1.64–2.74). The presence of 2 diseases was associated with a 2-fold increased rate of dementia (aHR 2.00, 95% CI 1.52–2.62). The specific constellation of comorbid NAFLD and stroke had the highest rate of dementia (aHR 3.04, 95% CI 1.61–5.74).

**Table 4:**
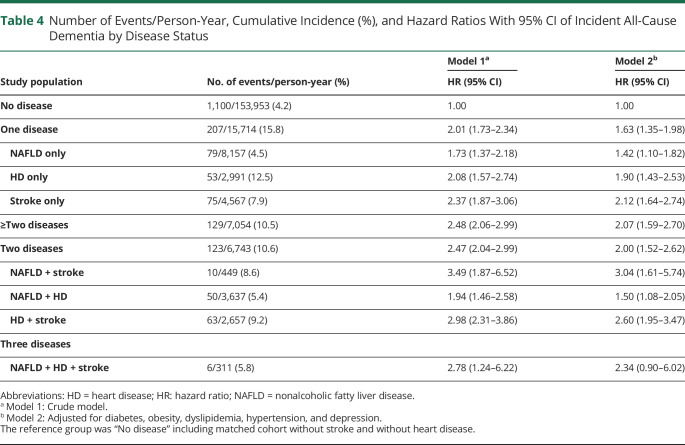
Number of Events/Person-Year, Cumulative Incidence (%), and Hazard Ratios With 95% CI of Incident All-Cause Dementia by Disease Status

### Sensitivity Analysis

Because dementia has a long prodromal phase, we analyzed the association among study participants with at least 5 years of follow-up time to address the concern of reverse causality. By doing so, we could also minimize any surveillance bias that might occur if patients with NAFLD were more likely to have dementia detected due to frequent contact with health care. The results of this analysis were similar in magnitude to our main findings (eTable 3, links.lww.com/WNL/C106)*.* We also excluded patients with cirrhosis at baseline to rule out the potential impact of hepatic encephalopathy misclassified as dementia. These results were also similar to our original findings with no meaningful differences (eTable 3)*.*

### Classification of Evidence

This study provides Class II evidence that NAFLD is associated with incident vascular and nonvascular dementia.

## Discussion

In this large matched cohort study of individuals aged 65 years and older, we found that NAFLD was associated with an increased rate of all-cause dementia, with some evidence suggesting that the main effect was due to vascular dementia. Comorbid cardiovascular diseases (e.g., NAFLD with stroke or heart disease) seemed to exacerbate the impact of NAFLD on dementia risk.

Although mounting evidence has suggested a link between NAFLD and cognitive dysfunction, the risk of dementia in patients with NAFLD has not been widely investigated.^9^ In our study, NAFLD was associated with a 38% higher rate of dementia. We present cumulative incidence data that can be important when communicating risk with patients and allocating healthcare resources.

The association observed in our study is consistent with a recent large Korean study of approximately 6 million individuals aged 40 years or older, which indicated an association between NAFLD and developing dementia.^25^ However, a study using primary care data from Germany suggested that NAFLD was not associated with dementia risk among adults aged 65 years or older.^16^ The disparity in results might be explained by the difference in the age distribution of the study populations, study settings, NAFLD diagnostic tools, and different confounders adjusted in the models. Furthermore, these studies all have a relatively short follow-up less than 8 years, leaving the results open to the influence of dementia's long prodromal phase. To this end, a previous study with a 20-year follow-up from our group found that the risk of dementia was not increased in biopsy-proven NAFLD.^15^ However, this study was underpowered to examine the effect of the comorbidities available in this study and those who underwent liver biopsy might receive increased surveillance and better treatment of contaminant cardiovascular risk factors. Nevertheless, a study by Weinstein et al.^26^ reported that reduced brain volume corresponding to 4 years of brain aging was independently related to NAFLD, suggesting that NAFLD is an emerging driver of cognitive aging.

Notably, we observed a difference in age at diagnosis in the association between NAFLD and incident dementia, with higher rates of dementia seen in patients diagnosed with NAFLD younger than age 85 years, while no significantly increased risk was found for those diagnosed older than age 85 years. We restricted the study population to those older than age 65 years because the mean age at dementia diagnosis is 85 years in Sweden^21^ and those with a first NAFLD diagnosis younger than age 65 years are less likely to develop dementia, given that the median follow-up time was 5.5 years in this study. The impact of NAFLD on dementia may be heterogeneous based on the age at NAFLD diagnosis, but studies that explicitly consider age at NAFLD diagnosis or NAFLD duration are scarce, primarily because NAFLD is often underdiagnosed. Nonetheless, studies examining the relationship between early age at diabetes onset and subsequent increased dementia risk may shed light on the plausible explanations, given that insulin resistance is a major feature shared by both patients with NAFLD and diabetes.^27,28^ Longer duration of hyperglycemia caused by insulin resistance may damage insulin signaling in the brain, leading to glucose neurotoxicity and accumulation of advanced glycated end products.^29^ The negative impact of chronic insulin resistance often seen in NAFLD may accumulate over time in patients who had NAFLD diagnosis at midlife, predisposing them to have a higher risk of dementia in late life. With a longer follow-up period, the magnitude of the associations reported here could presumably have been greater. We observed no increased dementia risk in patients with NAFLD diagnosed at age 85 years or older, partly because of the small difference in metabolic disorders between those with NAFLD and matched controls.^30^ Furthermore, the possibility of survival bias in this age group might play a role because older adults with severe NAFLD complications may have died early, leaving relatively healthy individuals in the risk set. Caution is needed when interpreting this result because the statistical power was limited due to the small number of patients aged 85 years or older.

Our results showed that NAFLD is associated with an increased risk of dementia, after adjusting for cardiovascular comorbidities. In addition, the presence of cardiovascular diseases further strengthened the risk of dementia. We also showed that NAFLD is associated primarily with vascular dementia, but not Alzheimer disease. These results are in accordance with the major hypothesis that the association between NAFLD and dementia is mainly driven by vascular damage in the brain. A direct link between NAFLD and subclinical vascular injuries, including atherosclerosis, arterial stiffness, and cerebral small vessel disease, was reported,^17,31^ and the clinical manifestation of vascular diseases, including stroke and heart diseases, is closely associated with vascular dementia.^18^ Beyond their already well-established individual association with incident dementia, we addressed their combined impact on dementia in old age by showing that dementia risk was found to increase >2-fold for patients with NAFLD comorbid with cardiovascular disease. Among all constellations, comorbid NAFLD and stroke and comorbid stroke and heart disease seem to be particularly damaging cognition. This finding might be partly attributed to increased surveillance in neurology clinics, i.e., stroke victims may receive dementia diagnosis earlier from physicians more alert to changes in cognition or functioning. Therefore, caution is needed when interpreting the magnitude of the results.

Alongside the vascular damage, other mechanisms have been suggested linking NAFLD to dementia. Hepatic dysfunction and insulin resistance may lead to insufficient amyloid clearance in the brain, and toxic metabolites produced in the injured liver may cross the brain-blood barrier leading to neuroinflammation that precedes pathology in the brain.^32,33^ Besides, it is recognized that liver fibrosis, rather than hepatic steatosis, is a strong prognostic factor for long-term complications such as cardiovascular diseases and mortality.^34,35^ In this regard, a previous study from our group demonstrated that histologic markers of fibrosis improved dementia risk prediction beyond that of conventional cardiometabolic risk factors. The advanced form of liver disease, such as fibrosis, may be required for cognition to be affected.^11^ To elucidate these mechanisms in greater detail, future studies of people with advanced fibrosis should integrate findings on cognitive functioning with neuropathologic and biomarker data.

Strengths of this study include a large population including all NAFLD patients aged 65 years or older and a matched cohort without NAFLD diagnosis identified from the NDR. Selection bias is minimal because physicians are required to report all medical data to the registers used, which are maintained by public institutions. The registers also allow for a long follow-up period, free from dropout apart from emigration. The limitations of this study also result from using the registers. Because NAFLD is often asymptomatic, it is often underdiagnosed despite a high prevalence in the general population. This misclassification of NAFLD may lead to an underestimation of the association between NAFLD and dementia. Furthermore, the NPR does not cover data from primary care, and hence, our results can only be generalized to patients in secondary or tertiary settings. That said, patients included in our study were diagnosed in specialty care and might represent more severe cases of NAFLD. Furthermore, dementia diagnosis might be misclassified as hepatic encephalopathy in patients with cirrhosis. However, our sensitive analysis, excluding all patients with cirrhosis, gave estimates similar to those of original analysis. The sensitivity of AD and VaD ascertainment from NPR is moderate,^21^ which might lead to falsely low estimates of the associations. In addition, we lack information on other possible confounders such as education, socioeconomic status, and cannot rule out the influence of residual confounding (i.e., duration of CVD).

In this large cohort study of older adults, NAFLD was associated with a higher rate of all-cause dementia. The finding was stronger among patients with both NAFLD and comorbid cardiovascular disease. These results highlight the possibility that targeted treatment of NAFLD and cardiovascular comorbidities may reduce the risk of dementia.

## Glossary

AD: Alzheimer disease
aHR: adjusted hazard ratios
CDR: cause of death register
CVD: cardiovascular diseases
HD: heart disease
HR: hazard ratio
ICD: International Classification of Disease
IQR: interquartile range
NAFLD: nonalcoholic fatty liver disease
NPR: National Patient Register
PPV: positive predictive value
